# Discovery of New Nitrite-Oxidizing Bacteria Increases Phylogenetic and Metabolic Diversity within This Niche

**DOI:** 10.1128/mBio.01619-18

**Published:** 2018-09-04

**Authors:** Douglas G. Capone

**Affiliations:** a Marine and Environmental Biology Section of Biological Sciences, University of Southern California, Los Angeles, California, USA

**Keywords:** Calvin cycle, Nitrotoga, chemoautotroph, nitrification, nitrite oxidation

## Abstract

K. Kitzinger et al. (mBio 9:e01186-18, 2018, https://doi.org/10.1128/mBio.01186-18) report the first isolation of a novel nitrite-oxidizing bacterium, “*Candidatus* Nitrotoga,” and provide the first detailed information on the physiology, phylogeny, and characterization of the nitrite-oxidizing system of this genus. The isolate was derived from a wastewater treatment system and exhibits adaptation and tolerance to relatively high levels of nitrite. The origin of its nitrite oxidoreductase is distinct from other known nitrite oxidoreductase (NXR) systems, having arisen either in this organism or by horizontal gene transfer. In contrast to many earlier-characterized nitrite oxidizers, it displays substantial metabolic plasticity in its mode of energetic metabolism with capabilities to use both hydrogen and sulfite as electron donors.

## Abstract (precis)

K. Kitzinger et al. (mBio 9:e01186-18, 2018, https://doi.org/10.1128/mBio.01186-18) report the first isolation of a novel nitrite-oxidizing bacterium, “*Candidatus* Nitrotoga,” and provide the first detailed information on the physiology, phylogeny, and characterization of the nitrite-oxidizing system of this genus.

## COMMENTARY

Since their first discovery and isolation by Sergei Winogradsky in the late 1800s, the nitrifying bacteria, including the ammonium oxidizers and nitrite oxidizers, have been an important focus of nitrogen cycle research in soils, freshwater, and marine systems (1). Winogradsky’s isolates were of the classical ammonium-oxidizing *Nitrosomonas* and nitrite-oxidizing *Nitrobacter* genera. Since those initial isolations, which confirmed the two-step process of complete nitrification that had been earlier suggested by Pasteur and others, our understanding of the phylogenetic and metabolic diversity of nitrifying organisms expanded only incrementally over the next century as researchers explored the diverse environmental niches that nitrifiers occupied (2). Most new ammonium oxidizers were identified in the beta- and gammaproteobacterial clades, while nitrite oxidizers occur in the alpha, beta, delta, and gamma clades of the *Proteobacteria* as well as in the *Nitrospira* phylum. The phylogenetic relatedness of these original isolates to other lithotrophic bacteria (e.g., sulfur- and hydrogen-oxidizing bacteria, as well as methanotrophic and methylotrophic organisms) was initially suspected based on ultrastructural, biochemical, and physiological evidence and subsequently confirmed as the molecular revolution of DNA sequencing studies progressed. We have also since implicated nitrifiers, and in particular ammonium oxidizers, in the production of nitrous oxide, a potent greenhouse gas (2).

However, along with advances in sequencing, microbial isolation techniques also have increased our understanding of the true phylogenetic diversity of nitrifiers and their physiology. The first major expansion of our knowledge of the real diversity of nitrifiers in the environment was the recognition in the early 1990s that large populations of the *Thaumarchaeota* in the sea and in soils were active ammonium oxidizers (3, 4). Around the same time came the discovery of the anaerobic oxidation of ammonium or the “anammox” pathway exclusively catalyzed by a group of the *Planctomycetes* bacteria (5).

Very recently, the classical “two-step” paradigm has been overturned by several groups by the discovery of complete ammonium oxidation to nitrate, or “comammox,” by certain *Nitrospira* species (6).

The genus “*Candidatus* Nitrotoga” within the betaproteobacteria has recently been implicated in the oxidation of nitrite through enrichment cultures from various environments, including tap water, eelgrass sediments, and permafrost soils, among others. Following in the footsteps of Winogradsky, Kitzinger et al. (7) now report the first isolation and characterization of a nitrite-oxidizing “*Ca*. Nitrotoga” obtained from a wastewater treatment plant. In contrast to some earlier enrichments with low-temperature optima, this isolate is a mesotroph with temperature optima between 24 and 28°C. Its *K*_*m*_ for nitrite is high relative to many other nitrite oxidizers, and it is tolerant of very high nitrite concentrations. Its nitrite oxidoreductase (NXR) is phylogenetically distinct from previously characterized forms in other nitrite oxidizers, including anammox bacteria, but it is not clear whether it arose in “*Ca*. Nitrotoga” or arrived from horizontal gene transfer.

“*Ca*. Nitrotoga” is chemoautotrophic with carbon acquisition through the classical Calvin-Benson-Bassham cycle, which, interestingly, it can power through a number of alternative means of energy generation in contrast to the limited metabolic plasticity of many known nitrite oxidizers. These include complete hydrogen and sulfite oxidation pathways.

The physiologically distinct characteristics of this new isolate with respect to its *K*_*m*_, relatively high optimal substrate concentrations, and diversity of electron acceptors indicate a much broader diversity of nitrite oxidizers in the environment and provide entrée into possible means of exploitation for waste treatment and other potential biotechnological applications.

Thus, the phylogenetic and metabolic diversity of nitrite-oxidizing bacteria is increased. The authors suggest that with further investigations, novel nitrite oxidizers will continue to be uncovered, adding to our knowledge of the amazing breadth of capabilities possessed by Earth’s greatest chemists, its microbial inhabitants.
